# Comparative study of imaging and pathology of primary mucinous adenocarcinoma with different imaging manifestations

**DOI:** 10.1111/crj.13750

**Published:** 2024-04-14

**Authors:** Jun Han, Jie Gao, Demei Chen, Mou Du, Yuxin Wu, Xidong Ma, Mei Xie, Hua Han, Chongchong Wu, Xinying Xue

**Affiliations:** ^1^ Department of Radiology Third Affiliated Hospital of Chongqing Medical University Chongqing China; ^2^ Department of Pathology Chinese PLA General Hospital Beijing China; ^3^ Department of Nuclear Medicine Chongqing University Cancer Hospital Chongqing China; ^4^ Department of Radiology, West China School of Public Health and West China Fourth Hospital Sichuan University Chengdu Sichuan China; ^5^ Department of Radiology Traditional Chinese Medicine Hospital of Changshou District Chongqing China; ^6^ Department of Respiratory and Critical Care Chinese PLA General Hospital Beijing China; ^7^ Department of Radiology Third Affiliated Hospital of Jinzhou Medical University Jinzhou Liaoning China; ^8^ Department of Radiology Chinese PLA General Hospital Beijing China; ^9^ Department of Respiratory and Critical Care, Beijing Shijitan Hospital Capital Medical University Beijing China

**Keywords:** adenocarcinoma, gene, imaging, mucus, pathological manifestation

## Abstract

**Background:**

Pulmonary mucinous adenocarcinoma is a special type of lung cancer. Its imaging manifestations are diverse, which brings challenges to clinical diagnosis. However, its formation mechanism is unclear.

**Objective:**

The objective of this study is to analyse the relevant mechanisms of the formation of pulmonary mucinous adenocarcinoma by observing its different imaging and pathological manifestations.

**Data and methods:**

Retrospective analysis was conducted on imaging manifestations and pathological data of 103 patients with pulmonary mucinous adenocarcinoma confirmed intraoperatively or pathologically.

**Results:**

Forty‐three patients had pulmonary mucinous adenocarcinoma with a solitary nodule/mass, 41 patients with localized pneumonia and 19 patients with diffuse pneumonia. Their CT manifestations included ‘falling snowflake sign’, ground‐glass opacity close to the heart, vacuous signs/honeycombing and withered tree branches. Under the microscope, all the three types of pulmonary mucinous adenocarcinoma had visibly formed mucus lakes but were made of tumour cells with totally different shapes, which included the goblet‐like shape (tall column‐like shape) and quasi‐circular shape. Tall column‐shaped tumour cells were negative or weakly positive for thyroid transcription factor‐1 (TTF‐1) and strongly positive for ALK mutation, whereas quasi‐circular tumour cells were positive for TTF‐1 and less positive for ALK mutation.

**Conclusion:**

The different imaging manifestations of mucinous adenocarcinoma are possibly due to the different amounts or viscosity of mucus produced, and the mechanisms of its formation may include (1) tumour cells in different shapes have different abilities to produce mucus; (2) tumours in different stages produce different amounts or viscosity of mucus; and (3) the TTF‐1 and ALK genes affect the production of mucus.

## Graphical abstract

In this work, retrospective analysis was conducted on imaging manifestations and pathological data of 103 patients with pulmonary mucinous adenocarcinoma confirmed intraoperatively or pathologically. The different imaging manifestations of mucinous adenocarcinoma are possibly due to the different amounts or viscosity of mucus produced.

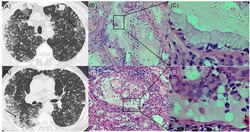

## 1 INTRODUCTION

Lung cancer is a neoplastic disease that causes the largest number of deaths, and one of its most common histological types is adenocarcinoma.^1^ Mucinous adenocarcinoma is a special subtype of lung adenocarcinoma, which accounts for approximately 5% of lung adenocarcinoma.^2^ Compared with other subtypes of adenocarcinoma, mucinous adenocarcinoma has special clinical manifestations, poor prognosis^3^, ^4^, ^5^ and various CT manifestations including ground‐glass opacity and nodular and flaky density. Some of its manifestations are easily confused with those of bacterial pneumonia. In the early stage of diagnosis and treatment, it is often misdiagnosed as pneumonia, and as a result, the best opportunity for treatment is missed. In some literatures, mucinous adenocarcinoma was reported to be classified based on CT manifestations into mucinous adenocarcinoma with a solitary nodule/mass, mucinous adenocarcinoma with localized pneumonia and mucinous adenocarcinoma with diffuse pneumonia.^6^, ^7^, ^8^, ^9^, ^10^ All these literatures presented analyses of the prognosis of mucinous adenocarcinoma versus non‐mucinous adenocarcinoma, but they did not explore the relevant mechanisms of the formation of mucinous adenocarcinoma. Therefore, to tentatively explain the mechanisms of the formation of different imaging manifestations of mucinous adenocarcinoma, this retrospective study was conducted by analysing the imaging manifestations and pathological data of 103 patients with pulmonary mucinous adenocarcinoma confirmed intraoperatively or pathologically, thereby providing a basis for the definitive diagnosis and treatment by the clinician in time.

## 2 DATA AND METHODS

### 2.1 Objects of study

This study retrospectively included 103 patients with pulmonary mucinous adenocarcinoma shown by multi‐slice spiral CT scans and ultimately confirmed intraoperatively (80 patients) or pathologically (23 patients) between September 2012 and July 2020 in the General Hospital of the People's Liberation Army, Beijing Shijitan Hospital, and the Third Affiliated Hospital of Chongqing Medical University. No evidence showed the presence of adenocarcinoma at other sites in patients. None of them had undergone radiotherapy or chemotherapy.

### 2.2 Imaging assessment

**FIGURE 1:**
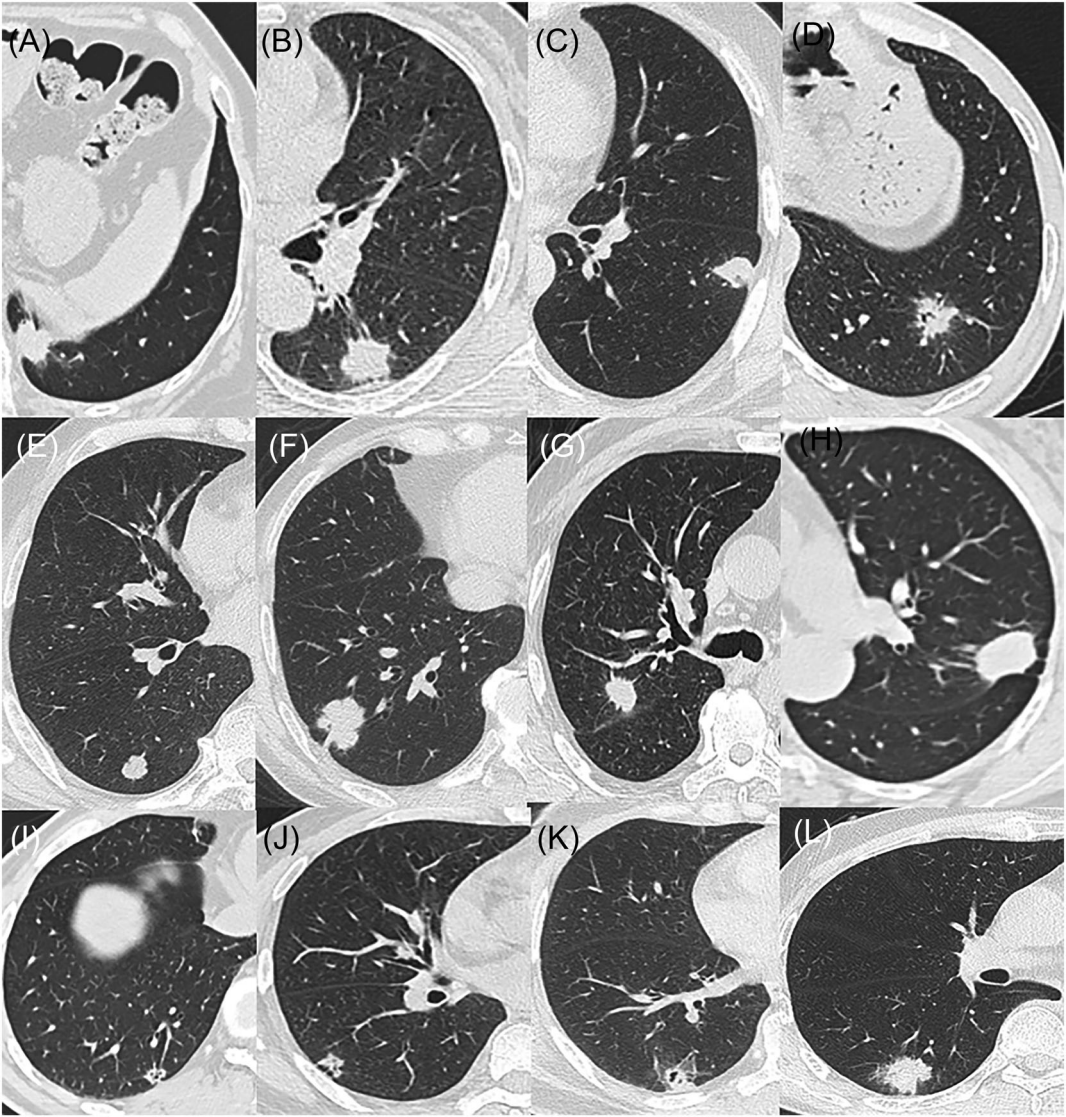
CT manifestations of pulmonary mucinous adenocarcinoma with a solitary nodule/mass with varying sizes. (A) The nodule was located in the posterior basal segment of the left lower lobe. (B) The nodule was located in the dorsal segment of the left lower lobe. (C) The nodule was located in the anteromedial basal segment of the left lower lobe. (D) The nodule was located in the basal segment of the left lower lobe. (E) The nodule was located in the posterior basal segment of the right lower lobe. (F) The nodule was located in the basal segment of the right lower lobe. (G) The nodule was located in the posterior segment of the right upper lobe. (H) The nodule was located in the tip‐posterior segment of the left upper lobe. (I) The nodule was located in the basal segment of the lower lobe of the right lung. (J) The nodule was located in the anterior basal segment of the right lower lobe. (K) The nodule was located in the posterior basal segment of the right lower lobe. (L) The nodule was located in the dorsal segment of the right lower lobe.

**FIGURE 2:**
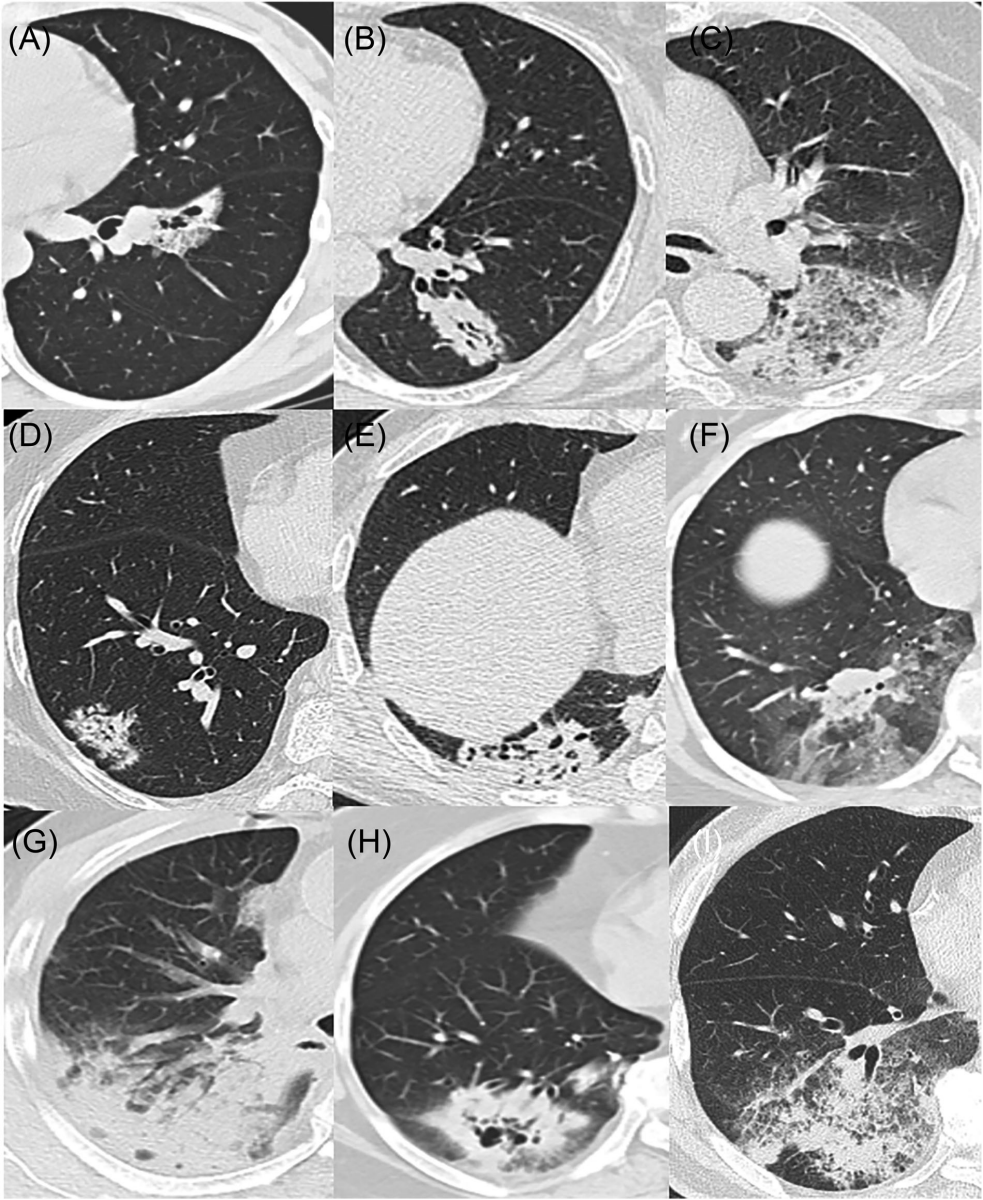
CT manifestations of pulmonary mucinous adenocarcinoma with localized pneumonia. (A) Patchy density was located in the anterior medial basal segment of the lower lobe of the left lung. (B) Patchy density was located in the posterior basal segment of the lower lobe of the left lung. (C) Patchy density was located in the dorsal segment of the lower lobe of the left lung. (D) Patchy density was located in the basal segment outside the lower lobe of the right lung. (E) Patchy density was located in the posterior basal segment of the lower lobe of the right lung. (F) Patchy density was located in the basal segment inside and posterior basal segment of the lower lobe of the right lung. (G) Patchy density was located in the posterior segment of the right upper lobe. (H) Patchy density was located in the posterior basal segment of the right lower lobe. (I) Patchy density was located in the lateral basal segment and posterior basal segment of the right lower lobe.

**FIGURE 3:**
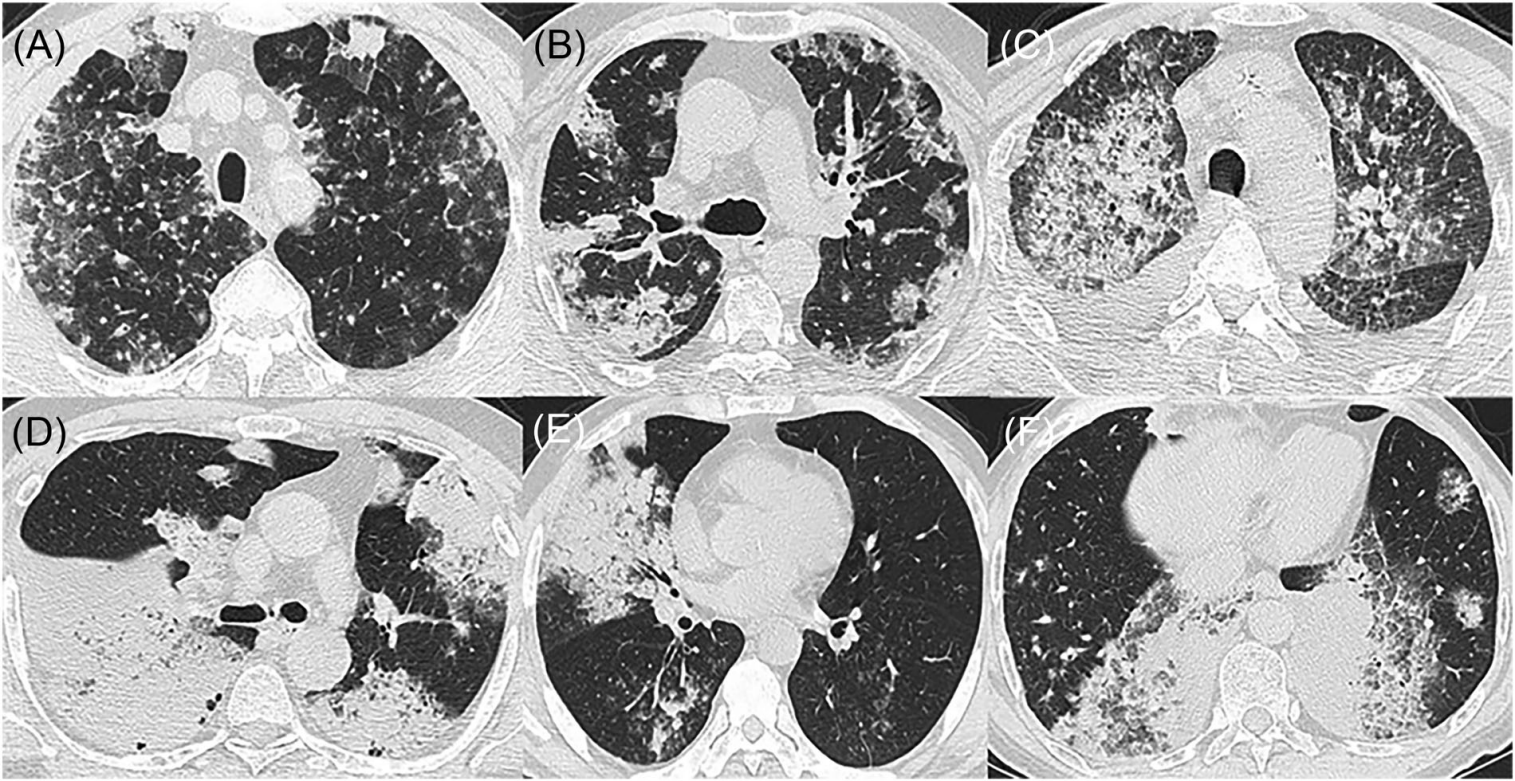
CT manifestations of pulmonary mucinous adenocarcinoma with diffuse pneumonia. (A) Patchy density was distributed in the upper lobe of both lungs. (B) Patchy density was distributed in the upper lobe of both lungs and the dorsal segment of the left lower lobe. (C) Patchy density was distributed in the upper lobe of both lungs and the dorsal segment of the left lower lobe, as well as bilateral pleural effusion. (D) Patchy density was distributed in the left and right middle lower lobes. (E) Patchy density was distributed in the right middle lobe and both lower lobes. (F) Patch density was distributed in the lower lobe of both lungs, mainly in the medial region, and in the lingual segment of the upper lobe of the left lung.

Based on the CT manifestations of pulmonary mucinous adenocarcinoma, we classified it into three types: (1) pulmonary mucinous adenocarcinoma with a solitary nodule/mass (only one nodule or mass and no lesions at other intrapulmonary sites; see Figure 1); (2) mucinous adenocarcinoma with localized pneumonia (a nodular lesion or mass accompanied by ground‐glass changes around; see Figure 2); and (3) pulmonary mucinous adenocarcinoma with diffuse pneumonia (a lesion with diffuse manifestations across pulmonary lobes or in both lungs; see Figure 3). Images (involving lesion margins and intralesional signs) were analysed by two associate chief physicians in radiology.

### 2.3 Pathological analysis

Lung tissue was prepared into paraffin‐embedded sections, stained and then microscopically observed and analysed by pathologists. Two morphologically different cell types from 30 patients were selected to undergo ALK gene testing, and two morphologically different cell types from 15 patients were selected to undergo high‐throughput sequencing of TTF‐1 (+) and TTF‐1 (−) specimens to seek differential gene expression patterns.

### 2.4 Statistical analysis

All statistical analyses were performed using SPSS (version 19.0). We adopted chi‐square test and variance analysis to evaluate the relationship between clinical features and classification. Fisher's exact test was used to evaluate the relationship between TTF expression and pathological types. Chi‐square was performed to assess the relationship between ALK mutation and pathological types. *P* < 0.05 was set to indicate a statistically significant difference.

## 3 RESULTS

### 3.1 Clinical characteristics and types of pulmonary mucinous adenocarcinoma on CT in patients

The 103 patients diagnosed with mucinous adenocarcinoma included 54 males and 49 females with a mean age of 59 years (range: 39–78 years). Forty‐four of them were smokers. Compared with patients with other types of pulmonary mucinous adenocarcinoma, those with diffuse pneumonia often have accompanying clinical symptoms. Of the 19 patients with pulmonary mucinous adenocarcinoma with diffuse pneumonia, 15 at visits had varying degrees of accompanying clinical symptoms including coughing and expectoration of sputum (Table 1).

**TABLE 1:** Clinical characteristics of patients with different types of pulmonary mucinous adenocarcinoma on CT

Characteristics	Solitary (*n* = 43)	Localized pneumonia (*n* = 41)	Diffuse pneumonia (*n* = 19)	Total (*n* = 103)	*P*
Sex					0.336
Male	19	24	11	54	
Female	24	17	8	49	
Age (years)					0.586
Mean	55.6 ± 11.0	59.6 ± 8.1	58.7 ± 9.3	57.8 ± 8.2	
Smoking history					0.517
Positive		18	10	44	
Negative	16	23	9	59	
Symptoms	27				0.000
Positive	11	25	15	51	
Negative	32	16	4	52	

### 3.2 CT manifestations and pathological types

**FIGURE 4:**
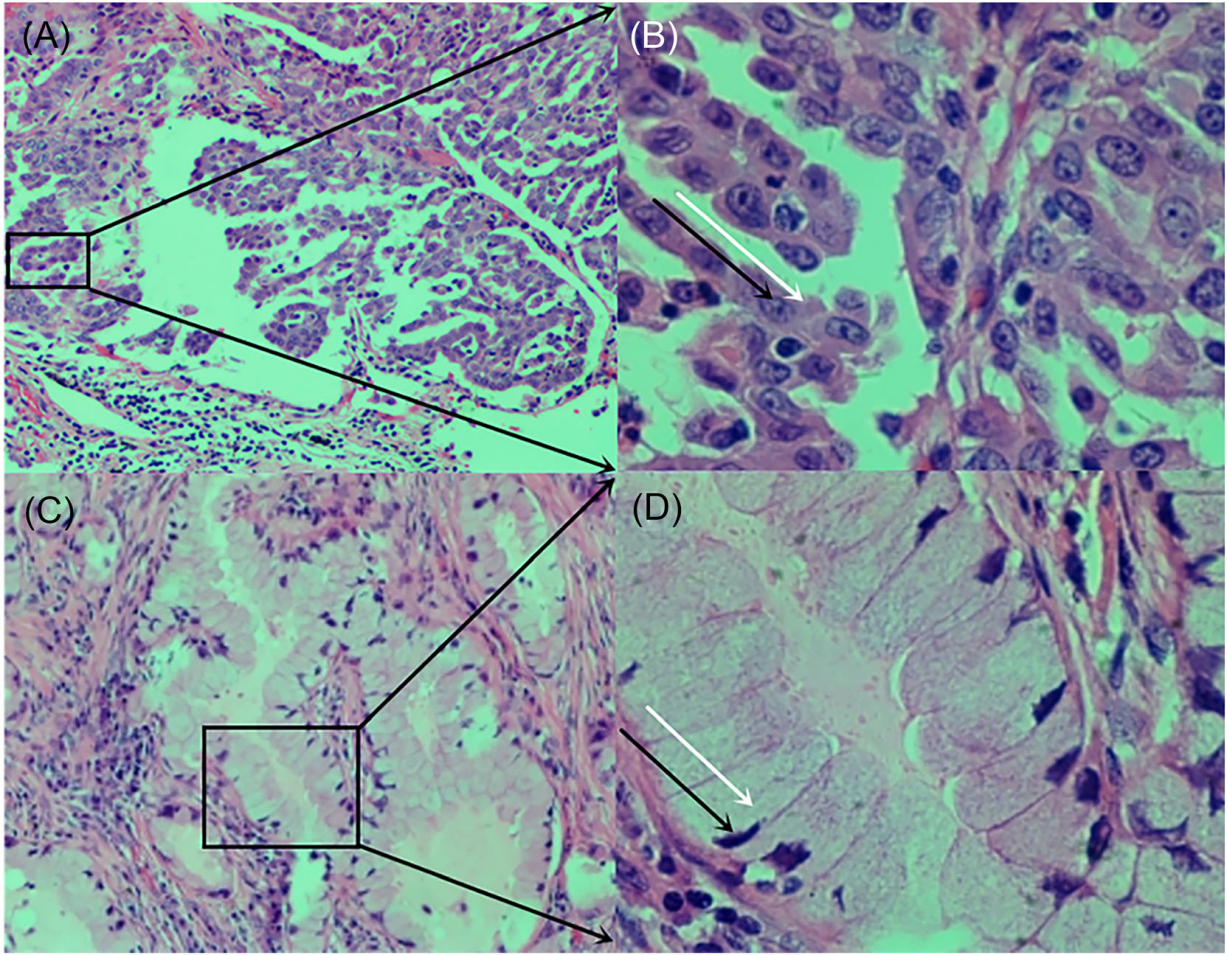
Morphology and characteristics of tumour under microscope. (A) Magnification 100X and (B) magnification 400X. Quasi‐circular tumour cells. The black arrow indicates tumour cells, and the white arrow indicates small amounts of thick mucus. (C) Magnification 100X and (D) magnification 400X tall column‐like tumour cells. The black arrow indicates tumour cells, and the white arrow indicates large amounts of thin mucus.

According to its imaging manifestations, pulmonary mucinous adenocarcinoma was classified into three types: pulmonary mucinous adenocarcinoma with a solitary nodule/mass (43 cases), pulmonary mucinous adenocarcinoma with localized pneumonia (41 cases) and pulmonary mucinous adenocarcinoma with diffuse pneumonia (19 cases). Pathological examination showed that the three types of pulmonary mucinous adenocarcinoma were made of tumour cells with the tall column‐like shape or quasi‐circular shape. Tall column‐like tumour cells had nuclei clinging to alveolar walls and column‐like cytoplasm that protruded out, whereas quasi‐circular tumour cells had large deeply stained nuclei with the quasi‐circular shape. Patients with pulmonary mucinous adenocarcinoma with localized pneumonia or diffuse pneumonia on CT usually had tumour cells with the goblet‐like shape (tall column‐like shape) shown by pathological examination. Microscopical observations showed that goblet‐like tumour cells had large amounts of thin mucus whereas quasi‐circular tumour cells produced small amounts of thick mucus (Figure 4).

### 3.3 Immunohistochemical analysis

**FIGURE 5:**
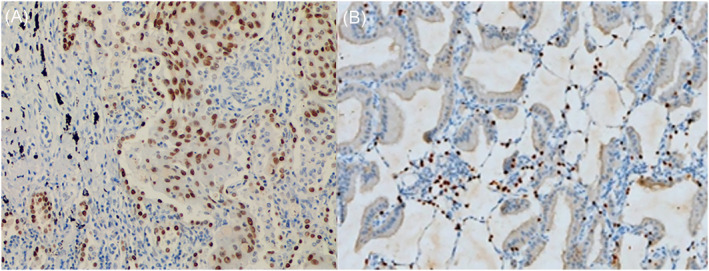
Positive (A) and weak positive (B) expression of TTF‐1

ALK gene testing showed that 13 of the 30 patients with tall column‐shaped tumour cells presented with mutation in the ALK gene, whereas 8 of the 30 patients with quasi‐circular tumour cells presented with mutation in the ALK gene (Table 2). Immunohistochemical analysis for TTF‐1 showed that 8 of the 15 patients with tall column‐shaped tumour cells showed a negative or weakly positive result for TTF‐1 (Figure 5), whereas all the 15 patients with quasi‐circular tumour cells showed a positive result (Table 3). The difference between the two patient populations with regard to their positive rate was statistically significant (*P* < 0.05).

**TABLE 2:** Relation between the ALK gene and the pathological morphology

Total (*n* = 60)	ALK gene		*P*
	(+)	(—)	
Tall column‐like shape (*n* = 30)	13	17	0.024
Quasi‐circular shape (*n* = 30)	5	25	

**TABLE 3:** Relation between TTF‐1 and the pathological morphology

Total (*n* = 30)	TTF		*P*
	(+)	(−)/(+/−)	
Tall column‐like shape (*n* = 15)	8	7	0.006
Quasi‐circular shape (*n* = 15)	15	0	

### 3.4 CT signs

**FIGURE 6:**
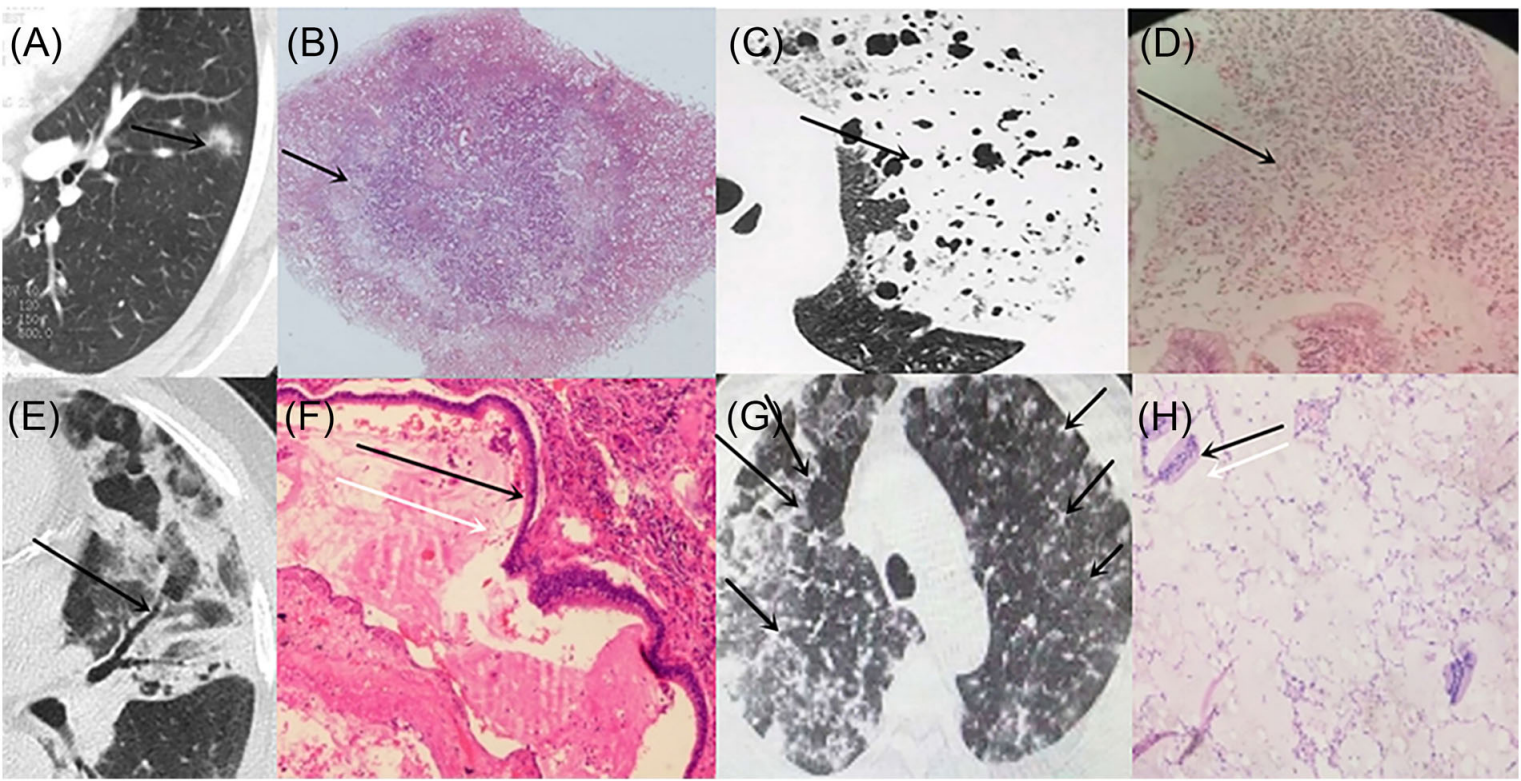
Comparison of CT findings and pathology. (A) The black arrow indicates ground‐glass opacity close to the heart and (B) H&E staining of lungmucinous adenocarcinoma tissue, magnification 100X, showed that there was more mucus close to the heart. (C) The black arrow indicates vacuous sign and (D) H&E staining of lungmucinous adenocarcinoma tissue, magnification 100X, showed nuclear fragmentation and mucus. (E) The black arrow indicates the air bronchogram and (F) H&E staining of lungmucinous adenocarcinoma tissue, magnification 100X; the black arrow indicates the wall of the branchus, the white arrow indicates the mucus. (G) The black arrow indicates ‘falling snowflake sign’ and (H) H&E staining of lungmucinous adenocarcinoma tissue, magnification 400X; the black arrow indicates the tumour cell, and the white arrow indicates the mucus.

Pulmonary mucinous adenocarcinoma with a solitary nodule/mass had non‐specific CT manifestations, that is, commonly visible signs including lobulation, spiculated sign, vascular convergence sign and pleural indentation. In contrast, pulmonary mucinous adenocarcinoma with localized pneumonia had specific CT manifestations, that is, ground‐glass opacity close to the heart, vacuous signs/honeycombing and withered tree branches. On CT, pulmonary mucinous adenocarcinoma with diffuse pneumonia manifested as ‘falling snowflake sign’, ground‐glass opacity close to the heart, vacuous signs/honeycombing and withered tree branches (Figure 6).

### 3.5 Imaging evaluation

**FIGURE 7:**
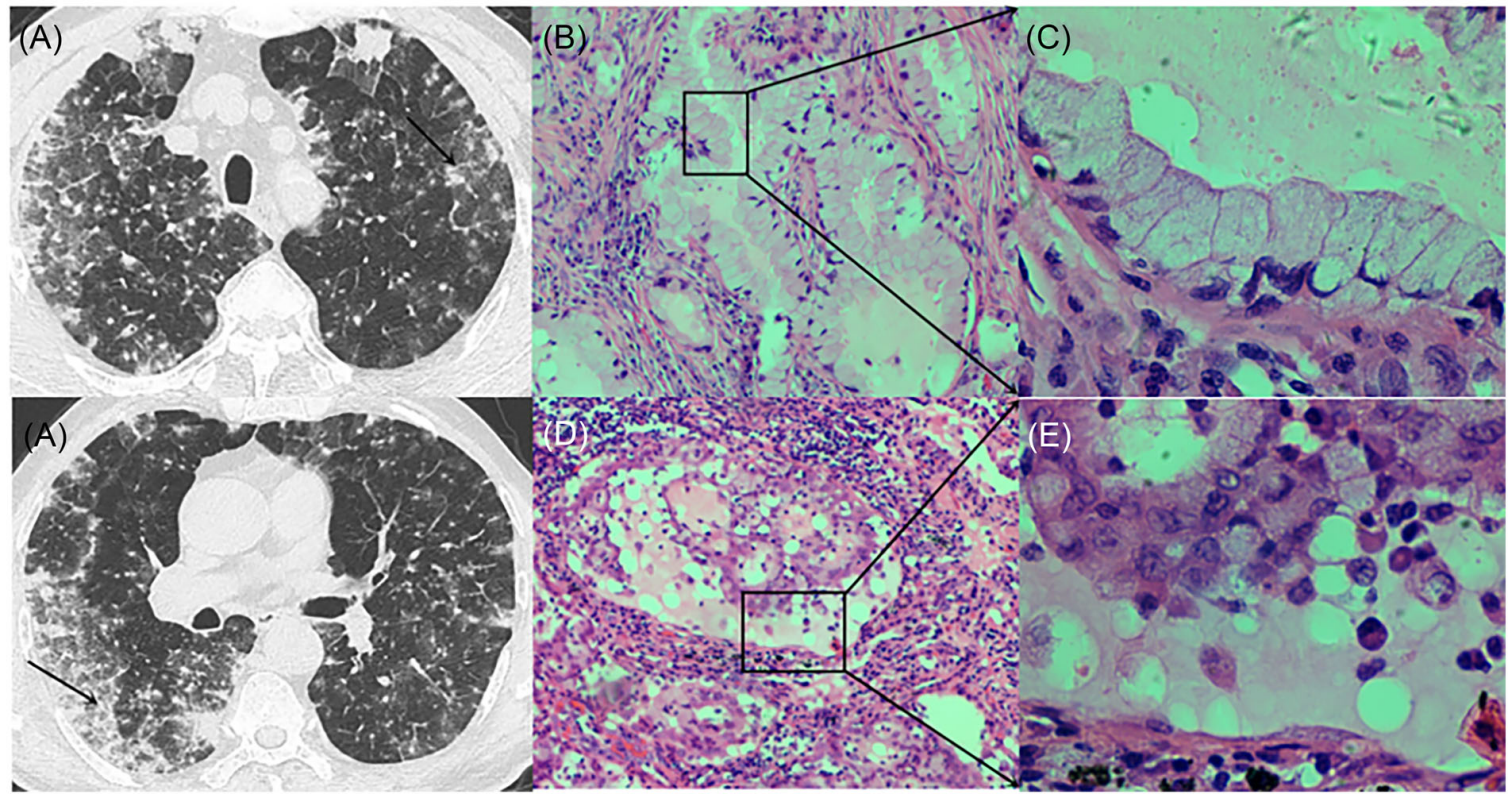
Two morphologically different cell types in the same patient. (A) The black arrow indicates two different biopsy sites in the same patient. (B) Magnification 100X. (C) Magnification 400X. (D) Magnification 100X. (E) Magnification 400X

A patient was admitted to hospital on the grounds of chief complaints of coughing and expectoration of sputum for 2 months, which were accompanied by fever for 1 week. Then, the patient was diagnosed with ‘pneumonia’. After admission, the patient coughed up a large amount of white sputum and had a body temperature of 38.0°C. A laboratory test showed a slightly elevated WBC count. A lung CT examination showed diffuse patchy shadows in both lungs. Bronchoscopic findings showed a large amount of mucus in the main bronchi. Microscopical observations of mucus taken randomly showed a large number of tumour cells. Later, biopsy of the left upper and lower lobes and the right lower lobe of the lung showed mucinous adenocarcinoma cells (Figure 7).

## 4 DISCUSSION

Pulmonary mucinous adenocarcinoma can have a variety of manifestations on CT, and it is the first time the mechanisms of its occurrence have been studied. This study showed that most patients with pulmonary mucinous adenocarcinoma with a solitary nodule/mass or localized pneumonia had no clinical manifestations, which was usually detected in medical examinations. In contrast, many patients with pulmonary mucinous adenocarcinoma with diffuse pneumonia presented with symptoms similar to those of pneumonia, including coughing and expectoration of sputum. At the initial visit, most cases of such type are prone to be misdiagnosed as pneumonia, and as a result, the best opportunity for treatment is missed. Previous studies have shown that mucinous adenocarcinoma had poorer prognosis than other types of adenocarcinoma.^3^, ^5^, ^11^, ^12^, ^13^ Thus, this study was performed to learn about the relevant mechanisms of the formation of mucinous adenocarcinoma and its development, thereby allowing the clinician to attach importance to its early detection, diagnosis and treatment. It was believed that the cause of the formation of mucus and its amount played a crucial role in the development of mucinous adenocarcinoma.^14^

**FIGURE 8:**
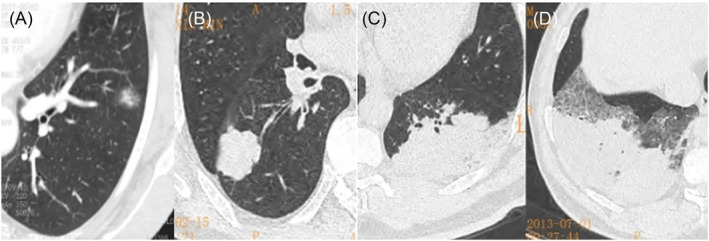
The boundary of mucinous adenocarcinoma. (A) A small lesion with an ill‐defined margin and (B) a large lesion with a well‐defined margin and (C, D) lesions with the same sizes but different margins

In this study, on CT, we found small lesions with ill‐defined margins and ground‐glass changes, large lesions with well‐defined margins and lesions of the same sizes with ill‐defined margins or well‐defined margins (Figure 8). Based on the pathological observations, we believed that the amount of produced mucus and its viscosity were the causes of its various CT manifestations. Then, we proposed the following three possible mechanisms of the formation of pulmonary mucinous adenocarcinoma. Mechanism 1: tumour cells in different shapes had different abilities to produce mucus. Pathological observations showed that tall column‐like tumour cells produced a large amount of thin mucus prone to be secreted outward and thus tended to have CT manifestations of focal or diffuse pneumonia. In contrast, quasi‐circular tumour cells produced a small amount of thick mucus; as a result, such mucus was localized and failed to be secreted outward, and then, it would take longer to form pulmonary mucinous adenocarcinoma with focal or diffuse pneumonia, and quasi‐circular tumour cells on CT frequently manifested as a nodule or mass, which was still localized with a well‐defined margin even it grew into a larger one. Some lesions had well‐defined margins on CT but still showed goblet‐like changes under the microscope. This might be because these lesions were in the early stage; that is, mucus produced by them had not been secreted outward yet. Mechanism 2: tumours in different stages produced different amounts of mucus or mucus with different viscosity. Pathological observations showed that a few lesions had both tall column‐like tumour cells and quasi‐circular tumour cells. Thus, we speculated that such two morphologically different cell types might change into each other due to the different stages the lesions were in. However, such mechanism still needs to be further confirmed. Mechanism 3: TTF‐1 and ALK genes (used for immunolabelling) had effects on the production of mucus. TTF‐1 was a nuclear protein mainly expressed in clara cells and type II alveolar epithelium.^15^ The immunohistochemical analysis for TTF‐1 in this study showed that most of tall column‐like tumour cells were negative or weakly positive for TTF‐1, whereas quasi‐circular tumour cells were positive for TTF‐1. It was believed that TTF‐1 might be expressed in mucinous adenocarcinoma. In a study on the correlation between TTF‐1 and the production of mucus in the lungs, Yutaka et al.,^16^ through mice experiments, found that positive TTF‐1 was expressed in non‐mucinous adenocarcinoma of the lung, whereas negative or weakly positive TTF‐1 was expressed in mucinous adenocarcinoma of the lung.^17^ TTF‐1 might have an effect on the production of mucus, that is, TTF‐1‐negative/weakly positive tall column‐like tumour cells were able to produce much more mucus. Previous studies showed that positive TTF‐1 was expressed in round cells and bronchial walls.^18^, ^19^ ALK gene testing also showed that two morphologically different cell types had different rates of mutation in the ALK gene: goblet‐like tumour cells has a significantly higher rate of mutation in the ALK gene than quasi‐circular tumour cells. Thus, the ALK gene is usually used as a marker for the diagnosis of mucinous adenocarcinoma.^20^ Previous studies found that the rate of mutation in the ALK gene in mucinous adenocarcinoma was higher than that of other types of adenocarcinoma,^21^, ^22^ and our study found a higher rate of mutation in the ALK gene in tall column‐like tumour cells. These findings indicate that the rates of mutation in the ALK gene are different in mucinous adenocarcinoma and non‐mucinous adenocarcinoma. Some studies demonstrate that the size of a lesion on CT is conductive to the evaluation of prognosis.^23^, ^24^ Of patients followed up by us, those with mucinous adenocarcinoma with diffuse pneumonia had a significantly lower 5‐year survival rate than those with the other two types of mucinous adenocarcinoma.

When a lesion shows on CT ground‐glass opacity close to the heart, vacuous signs/honeycombing, ‘withered tree branches’ or ‘falling snowflake sign’, possible mucinous adenocarcinoma should be highly suspected. When the lesion has nodular changes, the amount of secreted mucus will have an effect on its CT manifestations, and patients may manifest symptoms of pneumonia, including coughing, expectoration of sputum and even fever. In clinical diagnosis, the clinician should be highly alert to possible mucinous adenocarcinoma. It can be definitively diagnosed by biopsy and shall not be misdiagnosed as pneumonia; otherwise, the best opportunity for its treatment will be delayed.

## 5 CONCLUSION

Our study was aimed at having a better understanding of the mechanisms of the formation of mucinous adenocarcinoma with different CT manifestations. Pathological and immunohi‐stochemical data were combined to explore possible mechanisms of the formation of mucinous adenocarcinoma with different CT manifestations. However, a relevant further study should be performed to get a definitive answer. Radiomics can provide more important information, and we may consider using it in our future research.

## CONFLICT OF INTEREST

All authors declare no conflict of interest.

## AUTHOR CONTRIBUTIONS

Xinying Xue, Chongchong Wu and Hua Han designed the study, analysed the data and contributed to editing of the manuscript. Mou Du, Yuxin Wu, Mei Xie and Xidong Ma contributed to the implementation of the experiment. Jun Han participated in the experimental design and contributed to drafting the manuscript and revising it for intellectual content. Jie Gao was responsible for pathological analysis. Demei Chen analysed the data and conducted the experiments.

## ETHICS STATEMENT

The present study was conducted in compliance with the institutional policy regarding the protection of patient confidential information and was approved by the Research Ethics Committee of the third affiliated hospital of Chongqing Medical University (Chongqing, China; 2023 Research Ethics Review No. 83). All procedures were performed in accordance with the approved guidelines of the three hospitals.

## Data Availability

The datasets used during the current study are available from the corresponding author on reasonable request.
